# Supra-orbital Neuralgia

**Published:** 1882-11

**Authors:** J. E. Linnell

**Affiliations:** Norwich, Conn.


### SUPRA-ORBITAL NEURALGIA.

J. E. LINNELL, M. D., NORWICH, CONN.

*Editor* HOMŒOPATHIC PHYSICIAN:

The clinical case of Dr. Lippe in the July number of your journal so strongly reminds me of a case of supra-orbital neuralgia which came under my observation some time since, that I am strongly inclined to report it.

Mr. A., aged forty, had suffered most intensely from the above affection at times for a number of years, and had exhausted the skill of nearly all the allopathic physicians in the city. He had now been confined to his house for a number of weeks and had become exceedingly shattered nervously from the pain. At the time of the first visit the paroxysm had passed off and I could learn nothing definite in regard to it. I left him some Bell., with the promise of an early call the next A. M., as the paroxysms were periodical, occuring at five or six o'clock each morning and lasting for two or three hours. At my second visit I found him groaning with pain and hovering over the stove to keep warm. Every few minutes he would leave his seat by the stove and swing himself by clinching hold of an open door with both his hands, then he would resume his seat by the stove and call for a swallow of water. A dose of Ars.$^{200}$ relieved him at once, and he resumed his work in a few days.

A few months afterward he called at my office, saying he was threatened with another attack. He received another dose of Ars.$^{200}$ with a few blank powders. I have seen nothing more of the man. It is fair to presume he is cured of his neuralgia, as I am certain of his return to me if there was a recurrence of the disease, since he always failed to get relief elsewhere.

